# Performance evaluation of in-house developed Covid-19 IgG/IgM antibody rapid diagnostic kit

**DOI:** 10.1186/s13568-023-01620-0

**Published:** 2023-10-17

**Authors:** Vinaya Chandu Vidya Sagar G, PV Janardhan Reddy, Prashanth Suravajhala, Renuka Suravajhala, Uday Kiran V, Kavi Kishor PB, Venkateswarulu TC, Rathnagiri Polavarapu

**Affiliations:** 1 Genomix CARL Pvt. Ltd, Pulivendula, Andhra Pradesh 516 390 India; 2 Department of Biotechnology, Vignan’s Foundation for Science, Technology & Research Deemed to be University, Vadlamudi, Guntur, Andhra Pradesh 522 213 India; 3 Amrita School of Biotechnology, Amrita Vishwa Vidyapeetham, Clappana, Kerala 690525 India; 4 Bioclues.org, Hyderabad, India; 5 Department of Genetics, Osmania University, Hyderabad, Telangana 500 007 India

**Keywords:** Chemiluminescent immunoassay (CLIA), Clinical diagnosis, Antibody Rapid Diagnostic test kit, Infection, Serological tests

## Abstract

In the interest of preventing the Coronavirus Disease 2019 (COVID-19) pandemic from spreading, it is crucial to promptly identify and confine afflicted patients. Serological antibody testing is a significant diagnostic technique that is increasingly employed in clinics, however its clinical use is still being investigated. The present study was carried out to scrutinize how well Severe Acute Respiratory Syndrome Coronavirus-2 (SARS-CoV-2) antibody testing using in-house developed rapid antibody assay worked against the chemiluminescence (CLIA) assay. Either IgG positive (IgG + IgM-) or IgM positive (IgM + IgG-); both IgG and IgM positive (IgM + IgG+); and negatives (IgM- IgG-) have been evaluated. A total of 300 samples with diverse age and sexual identity data were included. The combined sensitivities for IgG + IgM+, IgM + IgG-, IgG + IgM- and IgG-IgM- were evaluated. More accurate diagnostic results may be obtained using molecular diagnostic tools. The Antibody Rapid Diagnostic kit’s (in-house developed) performance was satisfactory for determining the presence of Covid-19 infection with IgG and IgM positivity. The IgG and IgM positivity helped evaluate the immune response in the individual for the COVID-19 infection. These results lend support to the additional utilisation of serological antibody tests in the COVID-19 diagnosis.

## Key points

• We performed an archival research analysis to check Severe Acute Respiratory Syndrome Coronavirus-2 (SARS-CoV-2) antibody testing.

• We determined the presence of Covid-19 infection with IgG/IgM positivity.

## Introduction

SARS-CoV-2 was the unique Sarbecovirus that caused the pandemic Coronavirus disease 2019 (COVID-19), which had a significant influence on people’s health and wealth around the world (WHO, 2020). Although quantitative analysis of RNA byhigh performance liquid chromatography (HPLC) and evaluation by Reverse transcription digital PCR (RT-dPCR) for coronavirus RNA quantification have been introduced, the real-time reverse transcription polymerase chain reaction (RT-PCR) test detecting the viral nucleic acid through molecular testing is the gold standard (Alandijany et al. 2020). As COVID-19 makes a significant impact overall, and it will do so for years to come, it is crucial for nations to work together and be ready to respond to public health emergencies (Kang et al. 2021).

Immunoglobulin (Ig)A, IgG, IgM, or total antibodies that are directed against the SARS-CoV-2 specific spike protein (S) and/or nucleocapsid protein (N) are typically found in patient serum or plasma through serological testing (Fons and Krogfelt 2021). The host immune response and a crucial application of diagnostic tests are both informed by the kinetics of IgA, IgM, and IgG antibodies against the particular SARS-CoV-2 proteins (Pan et al. 2020; Paradiso et al. 2020). IgM antibody response is detectable as early as 3-days after illness onset, and peak levels were observed between the second and third week, while IgG antibody was detected from day four of illness, with peak levels being observed between the third and fourth week. Antibody profiling in COVID-19 patients has been described in several studies (Pan et al. 2020). In contrast, when IgM and IgG antibodies were profiled in 26 COVID-19 patients, either IgM or IgG emerged at the same time or the order of their appearance changed (Rosado et al. 2021). Another study found that IgA isotypes predominated within the first week of symptom onset when SARS-CoV-2 specific antibodies were longitudinally profiled in serum, saliva and bronchoalveolar lavage fluid (Harley and Gunsolus 2020). These findings might emphasise how crucial it is to diagnose acute COVID-19 by analysing all three isotypes (IgA, IgM, and IgG) (Imai et al. 2020). As per available data from COVID-19 patient follow-up studies, serum IgA and IgM antibodies gradually diminish after reaching peak levels, whereas IgG antibodies persist longer. According to recent findings, serological testing can identify infections a few days after symptoms first appear and may be an appropriate strategy to support molecular tests and improve the diagnostic accuracy (Qian et al. 2020). Moreover, serological tests might be helpful in developing nations with limited access to molecular testing (Rosado et al. 2021). Prior systematic reviews combined sensitivity stratified by test type and immunoglobulin class and found lower sensitivities with lateral flow immunoassays (LFIA) than enzyme linked immunosorbent assay (ELISA) and chemiluminescent immunoassay (CLIA) (Rosado et al. 2021).

Sincevirological testing offers the most convincing proof of the virus’s presence, to check SARS-CoV-2, it is frequently advised for the diagnosis of COVID-19 (Yadav et al. 2021). The gold standard diagnostic test recommended by current guidelines, reverse transcription polymerase chain reaction (RT-PCR), may identify SARS-CoV-2 RNA in respiratory samples (Qian et al. 2020). However, a number of variables, such as improper specimen collecting methods, viral load, time since exposure, and specimen source, have been documented to significantly alter RT-PCR assay performance, which may lead to false-negative test findings (Shamsollahi et al. 2020). Thus, there is an indefatigable need for more diagnostic tests (Xiang et al. 2020). As further diagnostic tools, serological assays for particular anti-SARS-CoV-2 antibodies, such as immunoglobulin M (IgM), immunoglobulin G (IgG), and immunoglobulin A (IgA) antibodies have been developed, they can reveal information regarding recent or previous infection. Nonetheless, high-quality data supporting the use of antibody tests in practice for COVID-19 are lacking, despite several studies reporting that they showed excellent sensitivity, ranging from 97.5 to 98.8% demonstrating increased diagnostic accuracy when paired with PCR (Qian et al. 2020). In fact, there were significant differences between studies in the antibody subtype, antigen included in the serological test kit, detection time, and measurement technique (Yangchun 2020). While some investigations only identified IgM or IgG individually, some studies detected both IgM and IgG and reported a positive result if either was present (Yadav et al. 2021; Zhou et al. 2021). On how to interpret the findings of an antibody test, there is disagreement (Zhou et al. 2021). The accuracy of a diagnosis may be impacted by the presence of IgM, IgG, either alone or in specific combinations, which may be connected to disease severity and immunization (Tan et al. 2021). As both IgG and IgM positives; only IgM positives; only IgG positives and IgG & IgM negatives were reported in all age groups under study for CLIA and rapid diagnostic test (RDT) respectively, this analysis sought to explore the diagnostic efficiency of SARS-CoV-2-specific antibodies stratified by these positive results (Yadav et al. 2021; Xiang et al. 2020). This analysis clarified the presence of the IgG and IgM antibody types, in contrast to earlier analyses that concentrated on the diagnostic efficacy of IgM + IgG+/-, IgG + IgM+/-, and IgM + or IgG+, which only provide hazy information (Tan et al. 2021).

## Materials and methods

### Study design

From patients at a tertiary care hospital who were thought to have COVID-19 infection, 300 blood samples were taken between the period April, 2021 to September, 2021. Only outpatient samples that arrived for CLIA analysis for quick test kit testing were chosen. Plasma was separated from the blood and stored at -20 °C until further use. The blood was collected into EDTA-coated vacutainer tubes, transferred to the lab, and centrifuged to extract the plasma. All procedures performed in studies involving human participants were in accordance with the ethical standards of the institutional ethics committee at Kurnool Medical College.

### Performance of CLIA

**Fig. 1:**
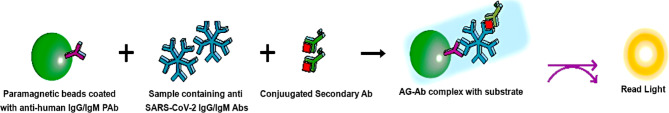
Procedure of Chemiluminiscence Immuoassay (CLIA)

For the purpose of identifying IgG and IgM, the chemiluminescence immunoassay (CLIA) was carried out using the fully automated iFlash 1800 (YHLO Biotech, China) chemiluminescence immunoassay analyser. Briefly, 20 L of serum or plasma were directly injected onto an autonomous platform using iFlash-SARS-CoV-2 IgG and/or oriFlash-SARS-CoV-2 IgM kits, as appropriate. In arbitrary units (AU) per millilitre, the reaction signal is indicated. IgM and/or IgG were deemed positive for the given sample if the threshold value exceeded 10.0 AU/mL (Fig. 1).

### Preparation of lateral flow assay cassette

**Fig. 2:**
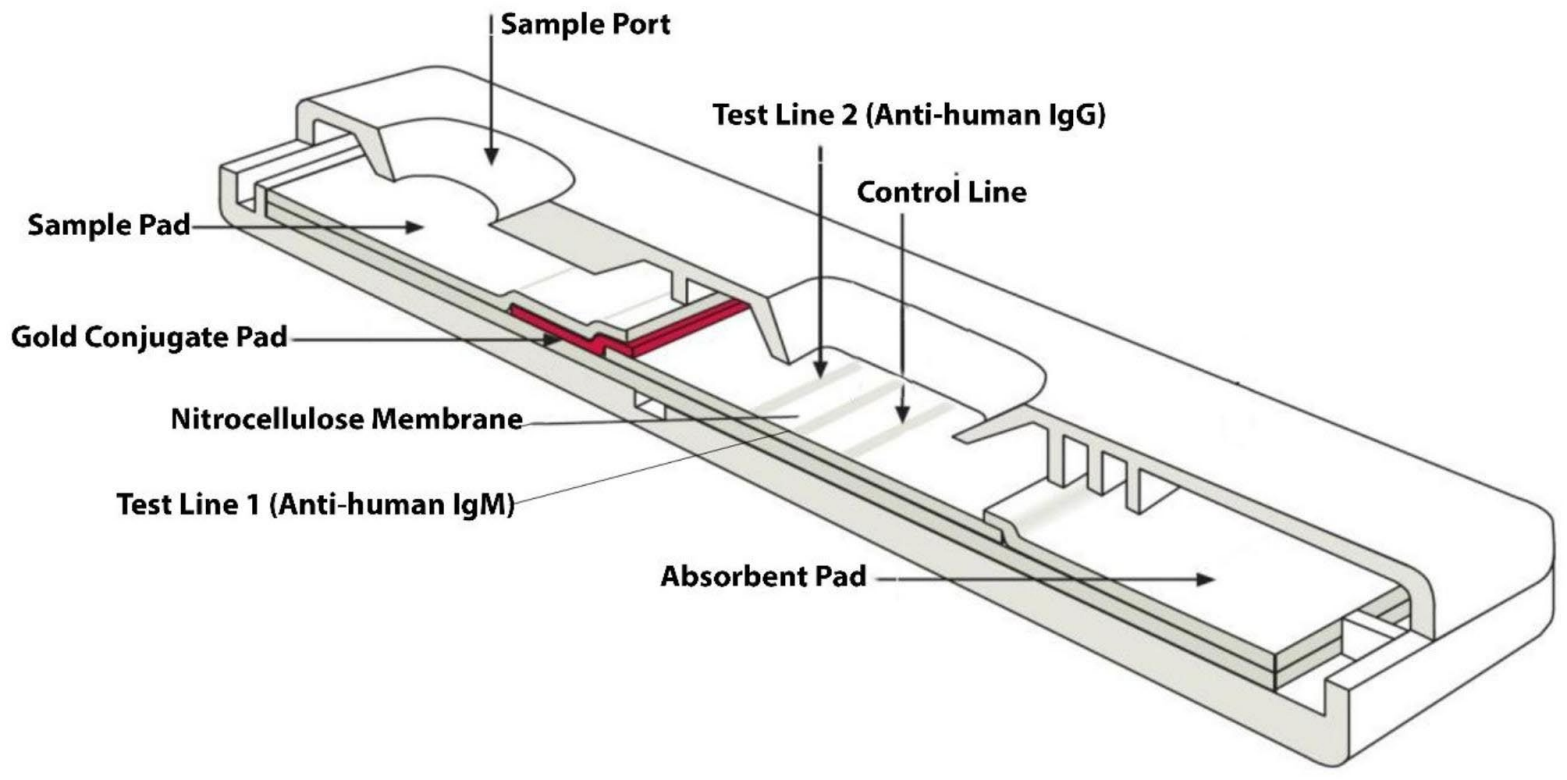
Layout of the Lateral Flow Cassette used for development of COVID-19 RDT kit

The lateral flow assay (LFA) device for rapid diagnostic test was developed internally (data not shown). On test lines 1 and 2, respectively, anti-human IgM and anti-human IgG monoclonal antibodies were coated, while biotinylated BSA was used as the control (C). The coated cards were made by adhering sample pads, conjugate cards with COVID-19 antigen coupled to gold nanoparticles (data not shown), nitrocellulose membranes with control and test lines, and absorbent pads to laminating sheets. The entire card is sliced to the required dimensions, which are then put into the plastic cassettes that have already been pre-moulded (by a third-party vendor), and finally pressed on a pressing machine. The final cassette is then sealed and placed into pouches filled with silica gel while being dried out (Fig. 2).

### Performance of lateral flow assay

The samples gathered above were subjected to the RDT 20 mL of whole blood or 10 mL of serum or plasma can be used for the experiment. During 20 to 30 s, the sample was introduced to the test device’s sample well. Then, 2 drops (90 μL) of the assay buffer were added. Within 15 min, the result was read. In as little as one minute, a strong positive specimen can generate a positive result.

### Interpretation of results from RDT

**Fig. 3:**
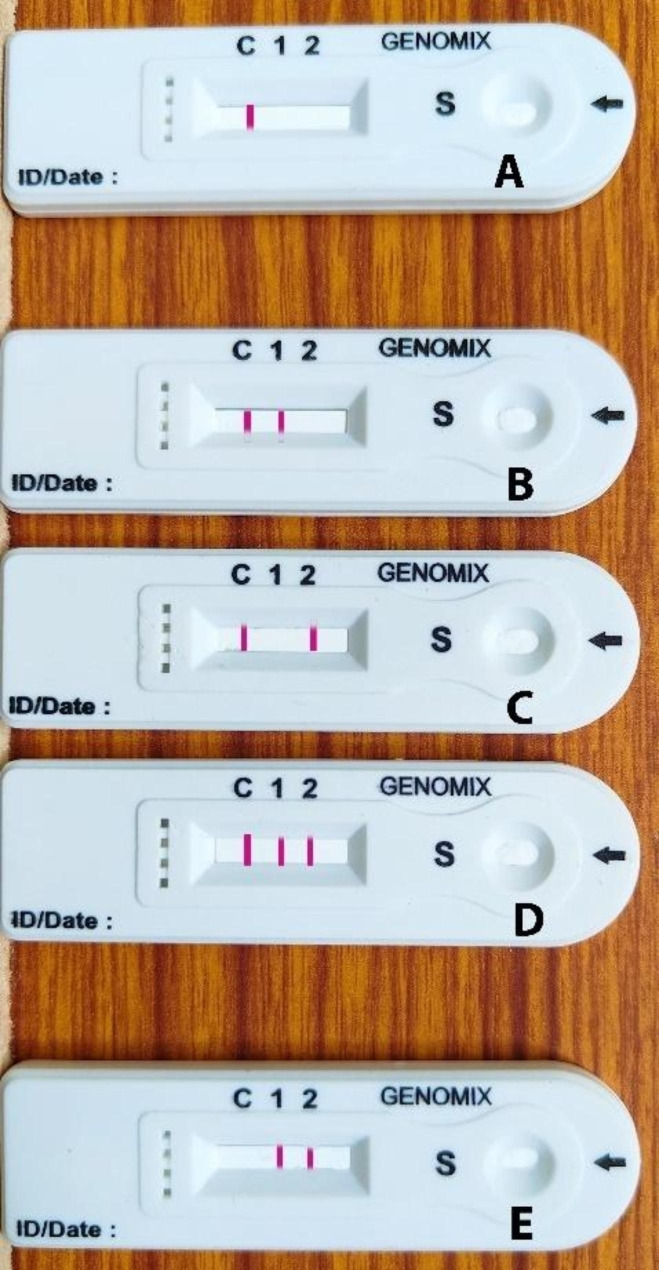
Figure showing the reading pattern of RDT kit. **A** – Negative; **B** – IgM Positive; **C** – IgG Positive; **D** – IgM & IgG Positive; **E** – Invalid

If pink bands develop at the control line (C) and/or one or more of the test lines, the sample is deemed to be positive for COVID-19 infection. If discernible bands are evident at test lines 1 and 2, then the sample is positive for both IgM and IgG. If the visible band only shows at test line 1, the sample is only IgG positive, and if it only appears at test line 2, the sample is only IgM positive. If the pink band appears in the control area (C), the sample is thought to be free of COVID-19 infection. The test is deemed unsuccessful if there is not any discernible band at the control region (C) (Fig. 3). With a different test gadget, the test must be repeated for the result.

### Data extraction

Data extracted from each study included the first author’s last name acronymed/anonymised, the age and sex of COVID-19 patients, the number of days since the onset of symptoms, the manufacturer of the test kit, the design of the study, the reference standard, the type of blood sample used in the RT-PCR, the methods, the antigen, and the types of antibodies used to detect antibodies. To build the 2–2 contingency table and evaluate sensitivity and specificity, true-positive, false-positive, false-negative, and true-negative findings were retrieved. This study aimed to explore the diagnostic efficacy of various antibody combinations, including IgG + IgM +, IgM + IgG-, IgG + IgM- and IgG- IgM- from CLIA and RDT, respectively. IgM + IgG-; IgG + IgM-; IgM + IgG +; IgM + IgG+/-; IgG + IgM+/-; and IgM + or IgG + combinations have been evaluated in age groups of 20–40, 40–60 and over 60 plus.

## Results

Using age and gender criteria, we investigated 300 samples. A total of 141 (47%) samples were from individuals above 60 years of age, 68 (22.67%) were between 40 and 60 years of age while 91 (30.33%) were between 20 and 40 years of age. From the 300 samples collected, 158 (52.66%) were from males and 142 (47.33%) were from females.

### Chemiluminescence immunoassay (CLIA) is comparable with RDT

#### Age based approach

**Fig. 4:**
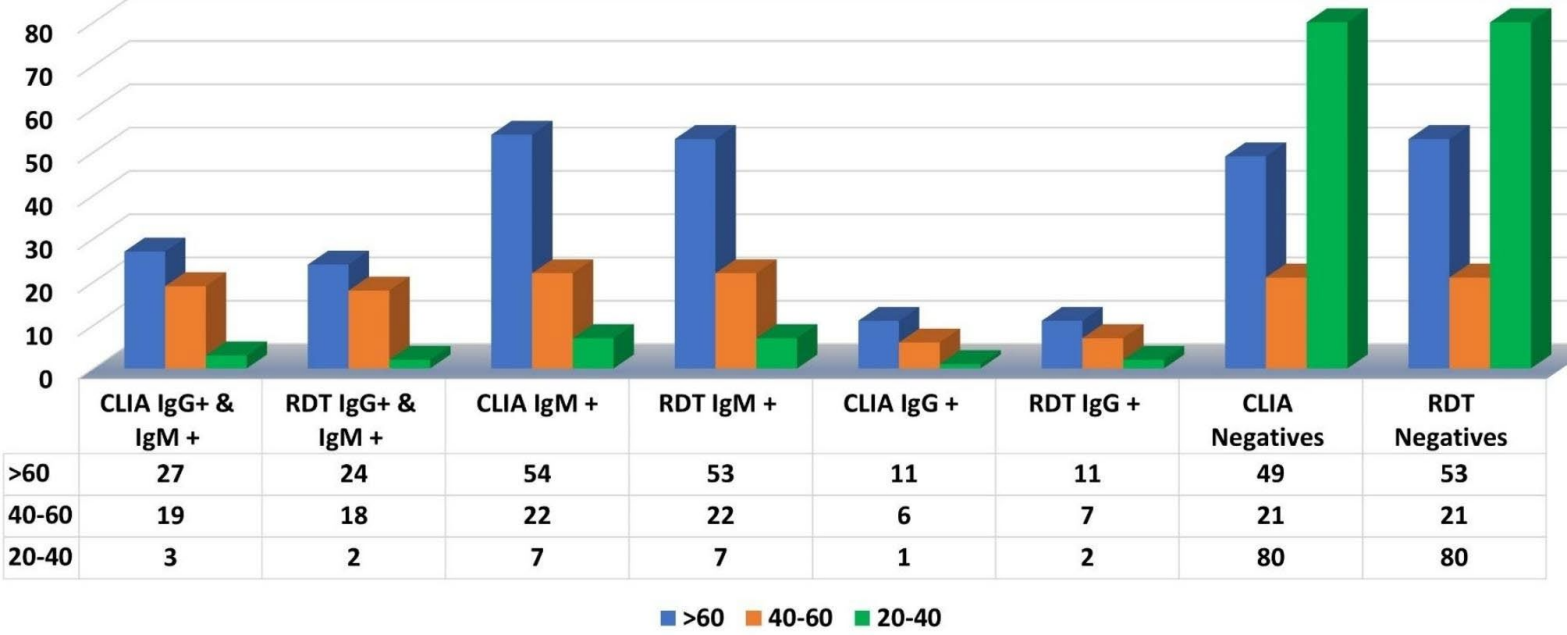
Graph showing the results of CLIA and RDT based on age groups

The findings indicated that 54 (38.29%) out of 141 samples (from > 60 years age group) tested by the CLIA were IgM + positive and 11 (7.8%) IgG + positive while 27 (19.14%) were found positive for both IgM + and IgG+ (Fig. 3). Moreover, 49 out of 141 negatives are present with a 34.75% respective prevalence. The results from RDT were found to be 53 (37.58%), 11 (7.8%), 24 (17.02%) positive for IgM+, IgG + and IgM+ & IgG + respectively while 53 (37.58%) were negative (Fig. 4).

IgG + IgM+, IgM +, IgG +, and negatives also exhibited 19 (27.94%), 22 (32.35%), 6 (8.82%) of positives, and 21 (30.88%) negatives respectively with reference to 40 to 60 year-old patients samples tested with 68 out of 300 samples from CLIA investigations. Similarly, from RDT investigations, 18 (26.47%), 22 (35.35%), 7 (10.29%) were positive for IgG + IgM+, IgM +, IgG + respectively and 21 (30.88%) were found negative (Fig. 4).

From the investigation of the samples from 20 to 40 year-old patients which were tested with 91 of 300 samples. CLIA results revealed a majority percentage of negatives i.e., 80 (87.91%), low percentages of positives i.e., 3 (3.29%), 7 (7.91%) and 1 (1.09%) positives were IgG + IgM +, IgM + and IgG + respectively. Same pattern was found from RDT results also. A total of 80 (87.91%) samples were negative while 2 (2.19%), 7 (7.69%) and 2 (2.19%) were positive for IgG + IgM+, IgM +, IgG + respectively (Fig. 4).

#### Gender based approach

**Fig. 5:**
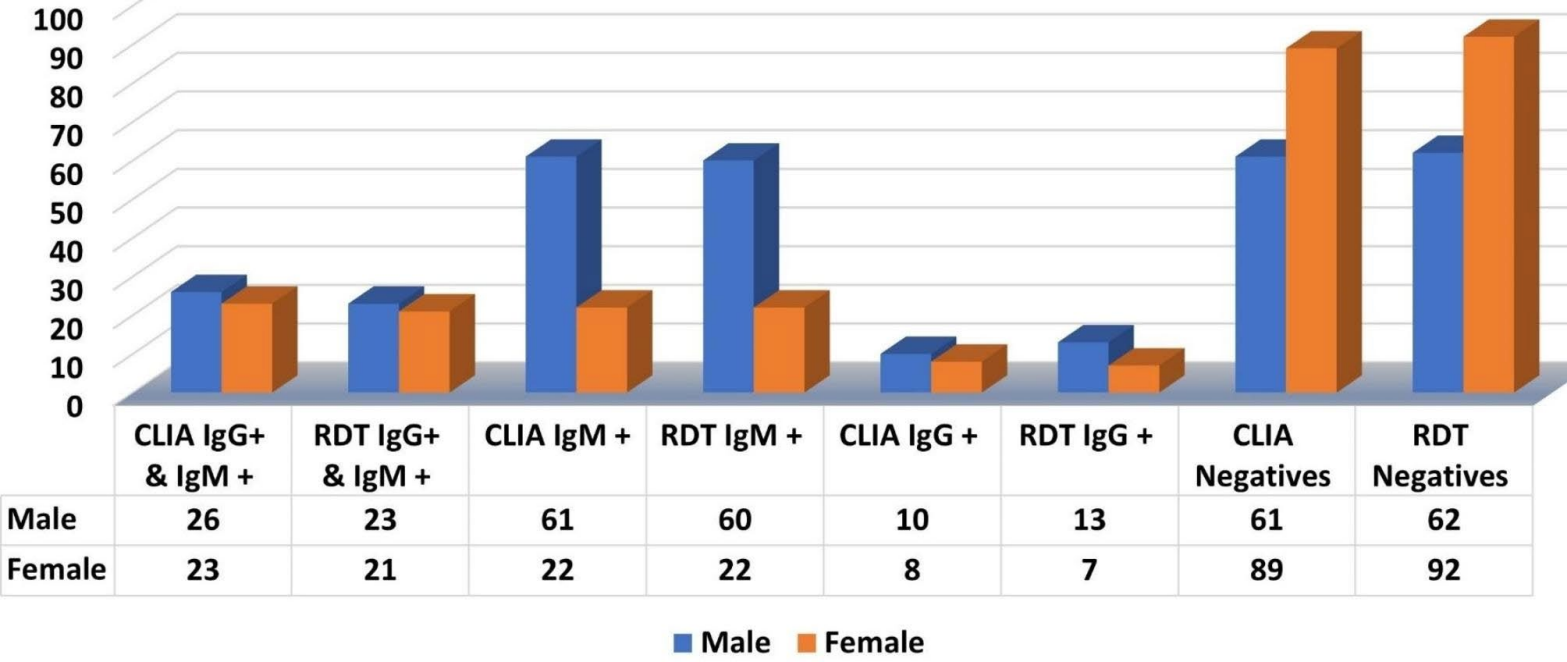
Graph showing the results of CLIA and RDT based on gender

From the results of CLIA and RDT, it was observed that males were more infected than females. The CLIA studies reported that 26 (16.45%) males and 23 (16.19%) females were positive for IgG + IgM + while RDT studies reported that 23 (14.55%) and 21 (13.29%) males and females respectively, were positive for IgG + IgM+. A total of 61 (38.60%), 10 (6.32%) males were positive for IgM + and IgG + respectively while 22 (15.49%) and 8 (5.63%) females were positive for IgM + and IgG + respectively from CLIA. From RDT studies, a total of 60 (37.97%) and 13 (8.22%) males were positive for IgM + and IgG + respectively while 22 (15.49%) and 7 (4.92%) females were positive for IgM + and IgG + respectively (Fig. 5).

CLIA studies reported 61 (38.60%) and 89 (62.67%) males and females respectively to be negative while RDT reported 62 (39.24%) and 92 (64.78%) males and females respectively negative (Fig. 5).

## Discussion

### Performance evaluation between CLIA and RDT

An internal COVID-19 IgG/IgM antibody fast diagnostic kit’s performance can be assessed by comparing it with a recognised reference method, such as CLIA. However, the evaluation should include the following parameters.

### Sensitivity

The test’s sensitivity is its capacity to accurately identify positive cases (Yangchun 2020). It is determined by taking the test’s true positive case detection rate as a proportion of all positive cases (Yangchun 2020). A set of positive samples were evaluated using both the quick diagnostic kit and the CLIA reference method in order to determine the sensitivity of the in-house created rapid diagnostic kit (RDT) (Zhou et al. 2021). The in-house developed RDT kit was found having a sensitivity of 97.33% against the CLIA test.

### Specificity

The capacity of a test to flawlessly identify negative cases is known as specificity. It is determined by taking the test’s proportion of truly negative cases out of all other negative cases (Dutta et al. 2020). A set of negative samples should be evaluated using both the quick diagnostic kit (RDT) and the CLIA reference method in order to assess the specificity of the in-house designed rapid diagnostic kit (RDT) (Yu et al. 2020). Thereafter, it is possible to compute and contrast the specificity of the fast diagnostic kit with the results obtained from the CLIA reference test. The in-house developed RDT kit was found having a specificity of 100% against the CLIA test.

### Accuracy

The capacity of a test to consistently detect both positive and negative cases is known as accuracy. It is determined as the proportion of genuine positive and true negative instances among all tested cases that were detected by the test (Grzelak et al. 2020). A set of positive and negative samples should be examined using the quick diagnostic kit and the CLIA reference method in order to gauge the accuracy of in-house designed RDT (Huyghe et al. 2020). The RDT kit developed was found to be having an accuracy of 98.67%.

### Precision

Reproducibility of test results is referred to as a precision. It is determined by measuring the variability of test findings obtained when the same sample is tested repeatedly (Huyghe et al. 2020). A set of samples should be evaluated frequently using the fast diagnostic kit to ascertain the accuracy of the in-house created RDT (Fong et al. 2020). For the tests done, the in-house RDT kit is having a precision of 1.0.

### Cross-reactivity

The test’s capacity to detect antibodies to different diseases that might be present in the sample is known as cross-reactivity (Dutta et al. 2020; Deeks et al. 2020). A set of samples having antibodies to additional pathogens should be evaluated using the quick diagnostic kit in order to ascertain the cross-reactivity of the in-house produced RDT (Yangchun 2020). The fast diagnostic kit’s cross-reactivity can then be juxtaposed to that of the CLIA reference method (Cheng et al. 2020). The effectiveness of the RDT was validated and its usefulness in detecting COVID-19 antibodies was assessed by comparing it to the CLIA reference kit (Cavalera et al. 2021). No cross-reactivity was found between different sample anti-sera (Dengue, chikungunya, Influenza A H1N1 and CMV) from both CLIA and RDT respectively.

This study has its limitations. First, the performance and interpretation of RDT may be end-user-sensitive and could have been duly used for venous blood samples (either plasma, serum or whole blood). However, our results from RDT could be vividly used to screen the latest XBB.1.116 variants as well. Although there are variances in sensitivity for various assay types, the antigen of choice did not seem to have an impact on the results of sensitivity. Our findings demonstrate that during the age and gender-based analysis, serological tests based on CLIA IgM-IgG and RDT IgM-IgG were equally sensitive. Because it takes time for an endogenous detectable antibody response to form, antibody detection has limits in error-free diagnosis, as was previously discussed. Nonetheless, serology could be useful in environments with limited resources if the results are interpreted while taking cognizance of the sensitivity’s limitations.

## Data Availability

Data related to this manuscript are available with VV and RP.
